# Multicenter dataset of multi-shell diffusion MRI in healthy traveling adults with identical settings

**DOI:** 10.1038/s41597-020-0493-8

**Published:** 2020-05-27

**Authors:** Qiqi Tong, Hongjian He, Ting Gong, Chen Li, Peipeng Liang, Tianyi Qian, Yi Sun, Qiuping Ding, Kuncheng Li, Jianhui Zhong

**Affiliations:** 1 Center for Brain Imaging Science and Technology, Key Laboratory for Biomedical Engineering of Ministry of Education, College of Biomedical Engineering and Instrumental Science, Zhejiang University, Hangzhou, Zhejiang China; 2 Beijing Key Laboratory of Learning and Cognition, School of Psychology, Capital Normal University, Beijing, China; 3 Beijing Key Laboratory of Magnetic Resonance Imaging and Brain Informatics, Beijing, China; 4 MR Collaboration NE Asia, Siemens Healthcare, Beijing, China; 5 MR Collaboration NE Asia, Siemens Healthcare, Shanghai, China; 6 Department of Radiology, Xuanwu Hospital, Capital Medical University, Beijing, China; 7 Department of Imaging Sciences, University of Rochester, Rochester, NY USA

**Keywords:** Brain, Magnetic resonance imaging, Diffusion tensor imaging

## Abstract

Multicenter diffusion magnetic resonance imaging (MRI) has drawn great attention recently due to the expanding need for large-scale brain imaging studies, whereas the variability in MRI scanners and data acquisition tends to confound reliable individual-based analysis of diffusion measures. In addition, a growing number of multi-shell diffusion models have been shown with the potential to generate various estimates of physio-pathological information, yet their reliability and reproducibility in multicenter studies remain to be assessed. In this article, we describe a multi-shell diffusion dataset collected from three traveling subjects with identical acquisition settings in ten imaging centers. Both the scanner type and imaging protocol for anatomical and diffusion imaging were well controlled. This dataset is expected to replenish individual reproducible studies via multicenter collaboration by providing an open resource for advanced and novel microstructural and tractography modelling and quantification.

## Abstract (summary)

**Table:** 

Measurement(s)	brain measurement
Technology Type(s)	magnetic resonance imaging (MRI)
Factor Type(s)	MRI scanner
Sample Characteristic - Organism	Homo sapiens

Machine-accessible metadata file describing the reported data: 10.6084/m9.figshare.12206378

## Background & Summary

Diffusion magnetic resonance imaging (MRI), a noninvasive technique for the exploration of brain microstructures, has been widely used in scientific research and clinical diagnosis. The quantitative measures from various diffusion models reveal different types of tissue viabilities in normal populations^1^ and pathologies in numerous diseases^2,3^. Over the last two decades, advances in MRI scanners have enabled the collection of diffusion data in clinically acceptable timeframes, rendering it an essential part of standard medical exams. To explore the neurobiological mechanism of the brain, a large number of advanced diffusion models and algorithms have been proposed to interpret the complexity of the brain microstructure by introducing more tissue compartments^4–7^, as well as revealing a more precise white matter fiber structure and connectivity from region to region^8–11^.

The promising applications of diffusion imaging in recent years have also inspired many multicenter collaborations to collect data from a large sample of subjects and to share the data with investigators all over the world to solve major scientific questions regarding the brain. Several multicenter collections involving diffusion imaging have been launched, including the Human Connectome Project^12^, the Adolescent Brain Cognitive Development study^13^, the Healthy Brain Network^14^, and the Alzheimer’s Disease Neuroimaging Initiative^15^, all of which have provided public access to the information therein. With multiple imaging centers sharing responsibility for the burdens (costs and manpower) of data collection, multicenter collaborations are effectively conducted to reduce the acquisition duration and to increase the diversity of the samples.

Unfortunately, one drawback of multicenter studies arises from the inevitable bias resulting from hardware and software differences among MRI scanners. These variances across scanners may reduce the reliability of the MR measures or even conceal the significance of the effect of interest^16–20^. However, it remains to be investigated whether the individual variances among scanners could be minimized by well-controlling the scanner hardware and software. An alternative way to improve data reproducibility would be retrospective harmonization^21–23^, which is efficient but requires more individual validation regarding different diffusion measurements. In both scenarios, data acquired from the same subjects in multiple scanners are required. Until now, only a few repositories have been available for obtaining diffusion data with scanners of different types^24^ and magnetic fields^25^. There is still a lack of diversity of multicenter data from strictly consistent acquisition.

Here, we released a multicenter diffusion dataset that was collected on three traveling subjects in ten centers and with two additional repeated scans in one center. Two features can be highlighted for the dataset. First, in all centers, the same type of scanners and same scan protocol were strictly followed, and the raw data were acquired by the same operating procedure and pre-processed with the same pipeline. Second, in the diffusion protocol, we used a popular multi-shell diffusion scheme, which can be adapted for most diffusion models either for multi-compartment analysis or for precise white matter tractography to resolve crossing fibers. Part of the dataset has been used successfully in one of our previous publications^26^. Generally, a well-established platform is crucial for the refinement and evaluation of novel methods. With highly equipped MRI scanners utilized in the data collection, this dataset could help in the evaluation of the individual reproducibility of advanced diffusion models for multicenter studies.

In the following sections, we briefly describe the dataset acquisition and processing procedures, technical validation, and sharing and access policy.

## Methods

### Data characteristics

The data were collected at 10 centers from October 2016 to November 2017. Three healthy traveling subjects (one male, 23 years of age, and two females, 26 and 23 years of age) were scanned in nine scanners once and in one scanner (Center 10) three times. All 10 scanners were 3 T MR MAGNETOM Prisma (Siemens, Erlangen, Germany), equipped with max gradient strength of 80 mT/m and slew rate of 200 T/m/s. The software version was Syngo MR VD13D in nine scanners and Syngo MR VE11C in one scanner (Center 09). In all scans, the same type of 64-channel head coil and the same acquisition parameters were used (details below).

The scans were processed by the same operator with a fixed operating procedure. For each subject, anatomical images and diffusion images were acquired. In the anatomical imaging, the field-of-view (FOV) was set according to the head orientation using the auto-align function in the Brain Dot Engine of the scanner, and in the diffusion imaging, the FOV orientation was set parallel to the anterior commissure-posterior commissure line. The FOV center was also aligned with the isocenter of the main magnetic field by moving the scan table.

### Anatomical image

T1-weighted anatomical images were acquired using a 3D magnetization-prepared two rapid acquisition gradient echo (MP2RAGE) sequence^27^. The imaging parameters were as follows: repetition time (TR)/echo time (TE) = 5 s/2.9 ms, inversion time (TI) = 700, 2500 ms, FOV = 211 × 256 × 256 mm^3^, voxel size = 1.2 × 1 × 1 mm^3^, bandwidth = 240 Hz/Px, generalized auto-calibrating partial parallel acquisition (GRAPPA) factor = 3, and the acquisition time was 8 minutes and 22 seconds.

### Diffusion-weighted image (DWI)

DWIs were obtained using a simultaneous multi-slice (SMS) spin-echo echo planar imaging (EPI) prototype sequence^28^. The imaging parameters were as follows: TR/TE = 5.4 s/71 ms, FOV = 220 × 220 mm^2^, slice number = 93, voxel size = 1.5 × 1.5 × 1.5 mm^3^, bandwidth = 1712 Hz/Px, GRAPPA factor = 2, and SMS factor = 3, with reversed phase-encoding (PE) directions along anteroposterior (AP) and posteroanterior (PA) separately. The diffusion duration and diffusion time were 15.9 ms and 34.4 ms, respectively, for the monopolar diffusion gradients. The diffusion scheme, containing 30 vectors with uniform angular coverage on each shell (b-values = 1000, 2000, and 3000 s/mm², non-colinear between any two shells), was generated from a multi-shell vector sampling tool^29^. Six non-diffusion frames were equally distributed in the scheme. The total acquisition time was 19 minutes and 04 seconds.

### Computational pre-processing

Practically, the DWIs acquired by MRI scanners are sensitive to noise, field inhomogeneity and head motion, which can cause image imperfections and require additional pre-processing for correction. In the released dataset, the raw images, stored in the Digital Imaging and Communications in Medicine (DICOM) format, were converted to the Neuroimaging Informatics Technology Initiative (NIfTI) format for pre-processing. The DWIs were pre-processed using a common pipeline, which included denoising using the MRtrix3^30^ (version 0.3.15-500-g382393bb), Gibbs-ring removal^31^, and distortion and motion correction using the FSL suite (version 5.0.11). Image distortion was estimated by the TOPUP tool from the non-diffusion images of PE directions to generate a field map function^32^. Both distortion and motion were jointly corrected with the EDDY tool^33^. In addition, the diffusion vectors were also rotated accordingly based on the EDDY correction. Finally, the images along the AP and PA directions were combined for subsequent analysis.

## Data Records

### Data privacy

Data collection was conducted with approval from the institutional review board of Xuanwu Hospital, Capital Medical University, Beijing, China, and all volunteers had signed the informed consent forms beforehand. Subject’s facial features had been removed from the images using the Freesurfer (version v1.379.2.73).

### Distribution for use

The dataset has been organized in the Brain Imaging Data Structure (BIDS) standard^34^ and is publicly available in figshare^35^. The multicenter scans of each subject are encoded as multiple sessions by labels identifying the center ID and repetition number. Each session folder contains the subfolders “anat” and “dwi” for anatomical and DWI images, respectively. The images are stored in a compressed NIfTI format, and the sidecar JSON files are listed together with the relevant images. In addition, a file with the suffix “eddy_parameters” produced by EDDY for each scan has also been provided under the folder “derivatives”.

## Technical Validation

### Quality control for diffusion data

The quality of the pre-processed images was evaluated. For each DW scan, we measured the signal-to-noise ratio (SNR) and ghost-to-signal ratio (GSR) from non-diffusion images. The background noise of all DWIs was also computed and compared. In addition, head motion was also estimated.

**Fig. 1:**
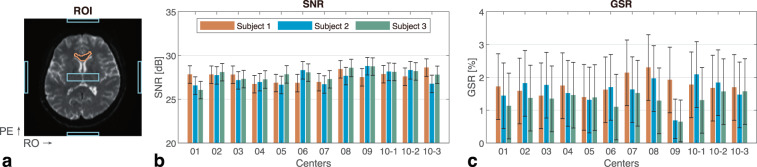
Quality metrics of the non-diffusion images. (**a**) The regions of interest (ROIs) for measuring signal-to-noise ratio (SNR) and ghost-to-signal ratio (GSR) are outlined in orange and blue, respectively. Phase-encoding (PE) and readout (RO) directions are marked. (**b**) The minimum, median, and maximum values of five SNR measures for each scan. (**c**) The minimum, median, and maximum values of six GSR measures for each scan.
- Head motion:
  The EDDY tool can estimate the head motion and eddy current-induced field when pre-processing the DWIs. The translation and rotation parameters relative to the first DWI frame were obtained as listed in *.eddy_parameters, together with 10 components interpreting the quadratic eddy current-induced field. In addition, two summarized volumetric movements were also generated as the root mean square (RMS) of the voxel displacement for each volume: one was calculated relative to the first frame, and the other was calculated between adjacent frames (as listed in *.eddy_restricted_movement_rms).
- Noise on DWI:
  The background noise of the DWIs was compared across centers. Four square ROIs of size 5 × 5 at the corners were drawn in the background, avoiding the Nyquist ghost region. The noise was calculated as the SD of all selected voxels divided by a correction factor of 0.66, accounting for the Rayleigh distribution on the image, as suggested in the NEMA standard^37^.

- SNR:
  The SNR was measured following the National Electrical Manufacturers Association (NEMA) standard on two non-diffusion images^36^. The mean values of two images within a white matter region of interest (ROI) at the genu of corpus callosum (GCC) were termed the signal. The noise was calculated from the standard deviation (SD) of the difference image within the same region divided by a correction factor of $\sqrt{2}$. In each scan, six non-diffusion images produced five SNR measures from every two adjacent images.
- GSR:
  The GSR is a measure of the Nyquist ghost artifact, which describes the signal leakage with a shift at 1/2 image size along the PE direction on image. In the non-diffusion images, a rectangular ROI at the center of the image of size 10 × 40 was selected as the signal region (ROI-s). Then, an ROI-g was defined as a pair of ghost regions by shifting the ROI-s half the image size up or down along the PE direction. Finally, an ROI-n was defined as two noise regions selected from the background with the same size as the ROI shifted left or right along the readout (RO) direction. These ROIs are outlined in blue in Fig. 1a. The mean intensities were computed within these defined ROIs. The GSR was calculated using the absolute signal difference between ROI-g and ROI-n and divided by the mean signal of ROI-s.

### Results of quality metrics

Figure 1 presents the quality metrics of the non-diffusion images. For the five SNRs and six GSRs calculated from each scan, their minimum, median, and maximum values are shown.

**Fig. 2:**
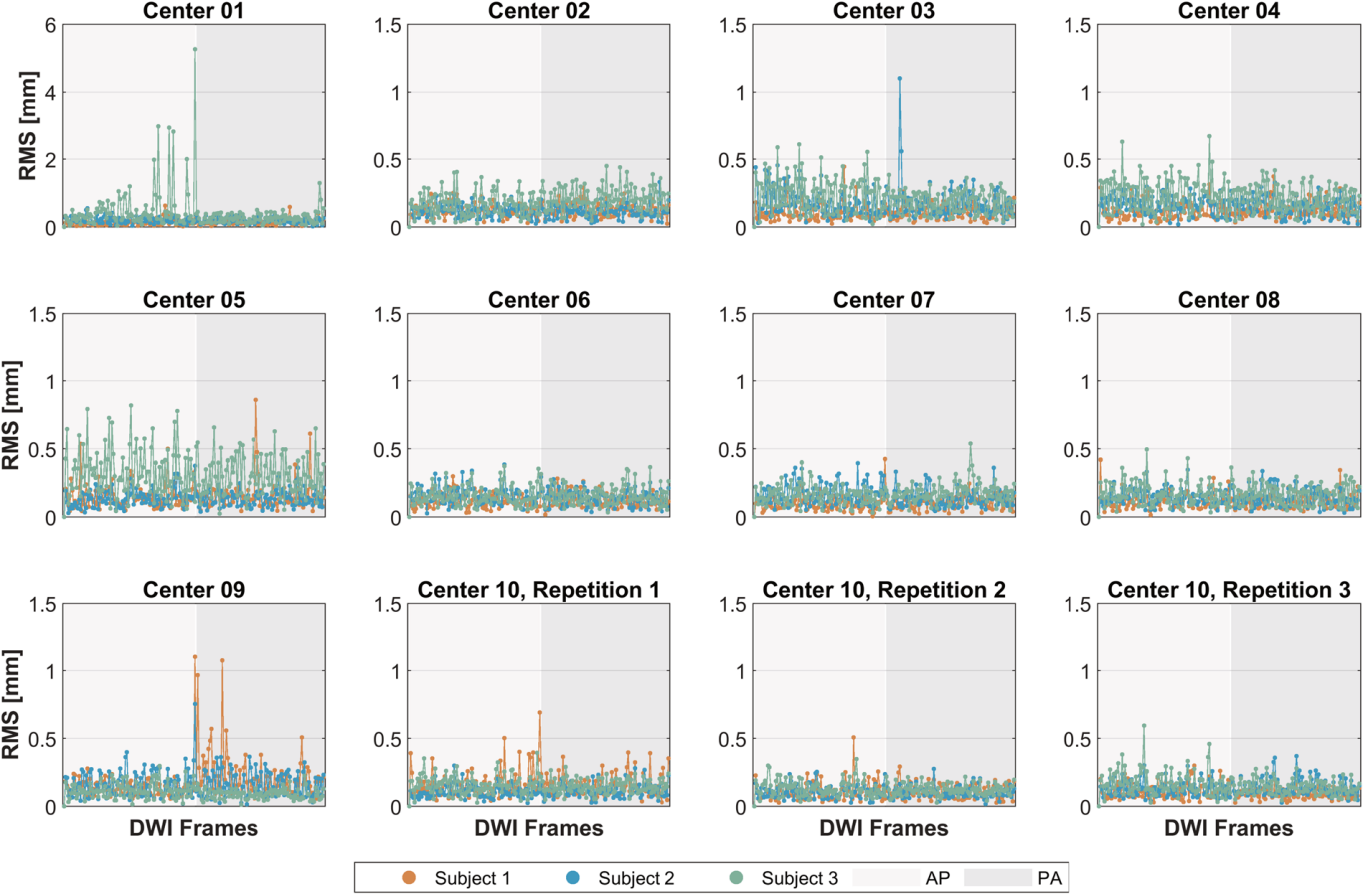
Motion measurement on diffusion-weighted images (DWIs) with PE directions along anteroposterior (AP) and posteroanterior (PA). The root mean square (RMS) to a previous frame is plotted.

Figure 2 presents the RMS motion metrics between adjacent DWI frames, including the AP and PA images.

**Fig. 3:**
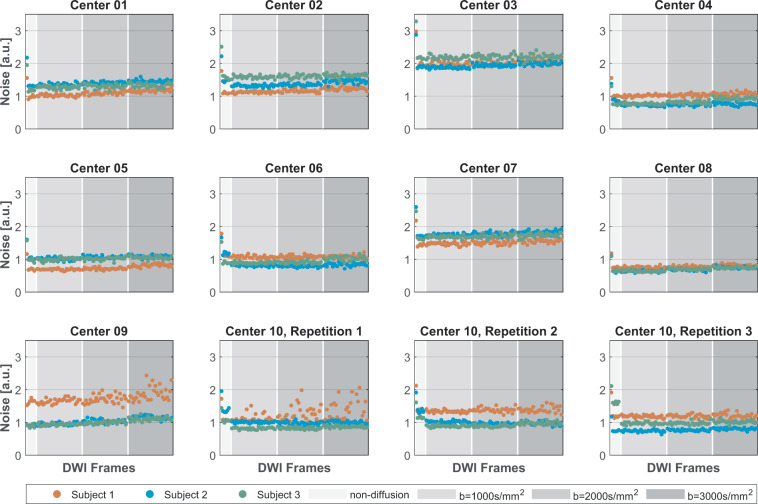
Noise measure on DWIs.

Figure 3 demonstrates the image noise evaluated from all DWI frames, which are reordered by b-values.

### Visualization and reproducibility of diffusion models

Beyond the quality metrics on the DWIs shown above, the reproducibility of diffusion outcomes generated by diffusion models or algorithms must also be considered. Here, we conducted a post-processing procedure using MRtrix3 for diffusion fiber tractography as an example. Since the framework of the fiber tracking algorithm was complicated, an intermediate measure reflecting voxel-wise fiber orientation together with the final tractography was selected for visualization. The intra-subject results were compared across all centers.

**Fig. 4:**
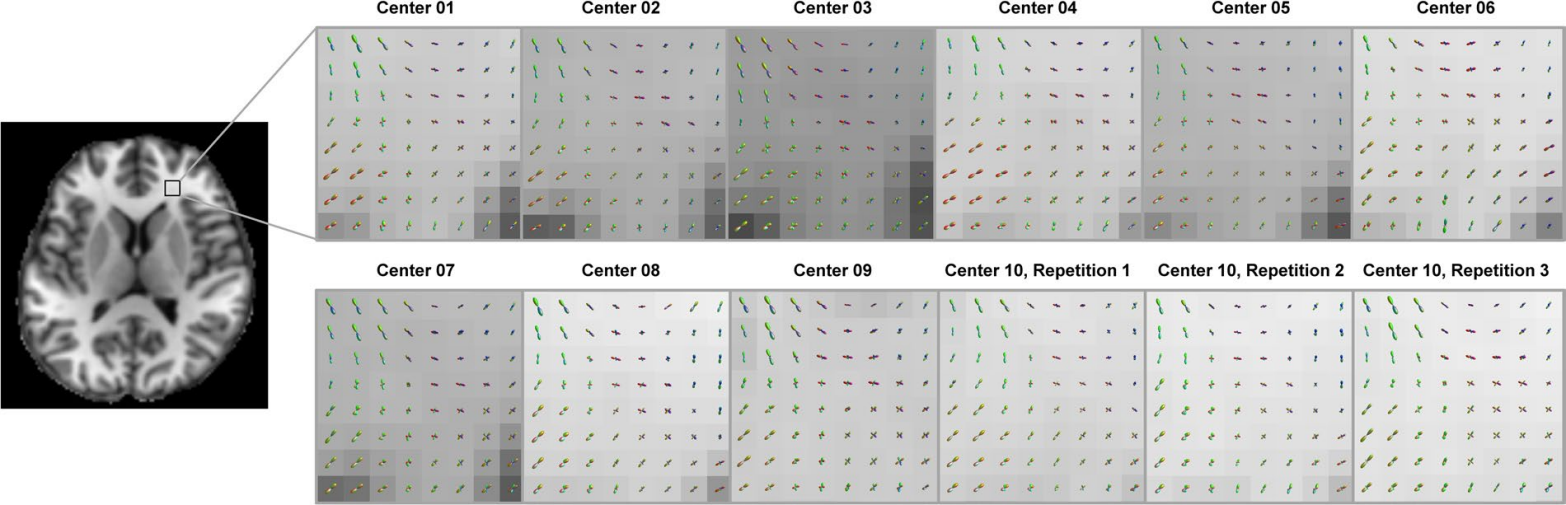
Fiber orientation distribution (FOD) from subject 1. The FODs are overlaid with registered T1-weighted images.
- Track density imaging (TDI):

- Fiber orientation distribution (FOD):
  The FOD represents the continuous distribution of underlying fibers for each voxel by the spherical deconvolution of the diffusion signal profile. To process the multi-shell diffusion data, multi-shell multi-tissue constrained spherical deconvolution^10^ was utilized to estimate the FOD with a maximum harmonic order of six. Figure 4 illustrates the FODs in an ROI containing crossing fibers that are mixed from the forceps minor, the anterior thalamic radiation, and the corticopontine tract.

**Fig. 5:**
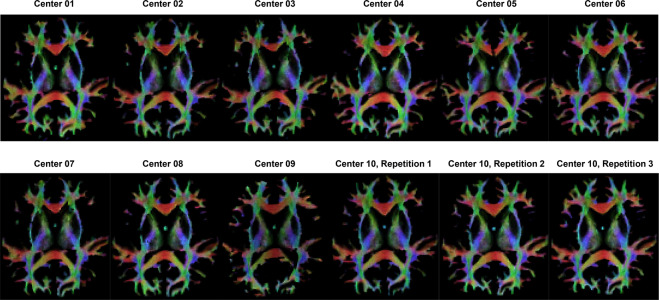
Track density imaging (TDI) from subject 1. The color is encoded by directions of fiber.

The anatomically constrained tractography algorithm^11^ was chosen for the multi-shell data to generate streamlines from the FOD with step size of 0.15 mm, length limitation from 3 to 250 mm and cut-off FOD amplitude of 0.06. Additionally, spherical deconvolution-informed filtering of tractograms was used to improve the accuracy of the fiber tractography^38^. After that, one million streamlines were reconstructed in the whole brain. To present the tractography, the TDI statistically measures the concentration of fiber streamlines within voxels. Figure 5 illustrates the TDI maps from all centers.

## Usage Notes

The data from eight centers [01–07 and 10] in the current dataset had been analyzed in our previous publications^26^, where they were denoted by A-H as follows: [01, D], [02, E], [03, A], [04, B], [05, G], [06, F], [07, C], and [10, H]. The data from centers 08 and 09 are newly added here. In addition, it should also be noted that we used an earlier version of the FSL suite (5.0.10) for pre-processing, and the Gibbs-ring removal was not used in our previous work^26^.

We also welcome any cooperation with us to fully explore this dataset.

## Data Availability

The codes for the pre-processing steps above were assembled in the released Dataset. We also shared a package of code for running the pre-processing pipeline and the technical validation, and can be accessed in figshare^39^.

## References

[1] Kodiweera C, Alexander AL, Harezlak J, McAllister TW, Wu YC (2016). Age effects and sex differences in human brain white matter of young to middle-aged adults: a DTI, NODDI, and q-space study. Neuroimage.

[2] Avants BB, Cook PA, Ungar L, Gee JC, Grossman M (2010). Dementia induces correlated reductions in white matter integrity and cortical thickness: a multivariate neuroimaging study with sparse canonical correlation analysis. Neuroimage.

[3] Faria AV (2010). Atlas-based analysis of neurodevelopment from infancy to adulthood using diffusion tensor imaging and applications for automated abnormality detection. Neuroimage.

[4] Zhang H, Schneider T, Wheeler-Kingshott CA, Alexander DC (2012). NODDI: practical *in vivo* neurite orientation dispersion and density imaging of the human brain. Neuroimage.

[5] Assaf Y, Basser PJ (2005). Composite hindered and restricted model of diffusion (CHARMED) MR imaging of the human brain. Neuroimage.

[6] Jespersen SN (2010). Neurite density from magnetic resonance diffusion measurements at ultrahigh field: comparison with light microscopy and electron microscopy. Neuroimage.

[7] Ferizi U (2015). White matter compartment models for *in vivo* diffusion MRI at 300mT/m. Neuroimage.

[8] Fan Q (2014). Investigating the capability to resolve complex white matter structures with high b-value diffusion magnetic resonance imaging on the MGH-USC Connectom scanner. Brain Connect..

[9] Maier-Hein, K. H. *et al*. The challenge of mapping the human connectome based on diffusion tractography. *Nat. Commun*. **8** (2017). 10.1038/s41467-017-01285-x PMC5677006 29116093

[10] Jeurissen B, Tournier JD, Dhollander T, Connelly A, Sijbers J (2014). Multi-tissue constrained spherical deconvolution for improved analysis of multi-shell diffusion MRI data. Neuroimage.

[11] Smith RE, Tournier JD, Calamante F, Connelly A (2012). Anatomically-constrained tractography: Improved diffusion MRI streamlines tractography through effective use of anatomical information. Neuroimage.

[12] Van Essen DC (2012). The human connectome project: a data acquisition perspective. Neuroimage.

[13] Casey BJ (2018). The adolescent brain cognitive development (ABCD) study: imaging acquisition across 21 sites. Dev. Cogn. Neurosci..

[14] Alexander LM (2017). An open resource for transdiagnostic research in pediatric mental health and learning disorders. Sci. Data.

[15] Weiner MW (2017). The Alzheimer’s disease neuroimaging initiative 3: continued innovation for clinical trial improvement. Alzheimer’s Dement..

[16] Fox RJ (2012). A validation study of multicenter diffusion tensor imaging: reliability of fractional anisotropy and diffusivity values. Am. J. Neuroradiol..

[17] Sasaki M (2008). Variability in absolute apparent diffusion coefficient values across different platforms may be substantial: a multivendor, multi-institutional comparison study. Radiology.

[18] Seo Y, Wang ZJ, Morriss MC, Rollins NK (2012). Minimum SNR and acquisition for bias-free estimation of fractional anisotropy in diffusion tensor imaging - a comparison of two analytical techniques and field strengths. Magn. Reson. Imaging.

[19] Teipel SJ (2011). Multicenter stability of diffusion tensor imaging measures: a European clinical and physical phantom study. Psychiatry Res.: Neuroimaging.

[20] Zhou X (2017). Quantitative quality assurance in a multicenter HARDI clinical trial at 3T. Magn. Reson. Imaging.

[21] Tax CM (2019). Cross-scanner and cross-protocol diffusion MRI data harmonisation: a benchmark database and evaluation of algorithms. Neuroimage.

[22] Karayumak SC (2019). Retrospective harmonization of multi-site diffusion MRI data acquired with different acquisition parameters. Neuroimage.

[23] Pohl KM (2016). Harmonizing DTI measurements across scanners to examine the development of white matter microstructure in 803 adolescents of the NCANDA study. Neuroimage.

[24] Ning, L. *et al*. Muti-shell diffusion MRI harmonisation and enhancement challenge (MUSHAC): progress and results. in *Computational Diffusion MRI* 217–224 (Springer, Cham, 2019).

[25] Sotiropoulos SN (2016). Fusion in diffusion MRI for improved fibre orientation estimation: an application to the 3T and 7T data of the human connectome project. Neuroimage.

[26] Tong Q (2019). Reproducibility of multi-shell diffusion tractography on traveling subjects: a multicenter study prospective. Magn. Reson. Imaging.

[27] O’Brien, K., Krueger, G., Lazeyras, F., Gruetter, R. & Roche, A. A simple method to denoise MP2RAGE. in *Proc. 21th International Society for Magnetic Resonance in Medicine* 0269 (Int. Soc. Magn. Reson. Med., 2013).

[28] Setsompop K (2012). Blipped-controlled aliasing in parallel imaging for simultaneous multislice echo planar imaging with reduced g-factor penalty. Magn. Reson. Med..

[29] Caruyer E, Lenglet C, Sapiro G, Deriche R (2013). Design of multishell sampling schemes with uniform coverage in diffusion MRI. Magn. Reson. Med..

[30] Veraart J (2016). Denoising of diffusion MRI using random matrix theory. Neuroimage.

[31] Kellner E, Dhital B, Kiselev VG, Reisert M (2016). Gibbs-ringing artifact removal based on local subvoxel-shifts. Magn. Reson. Med..

[32] Andersson JLR, Skare S, Ashburner J (2003). How to correct susceptibility distortions in spin-echo echo-planar images: application to diffusion tensor imaging. Neuroimage.

[33] Andersson JLRR, Sotiropoulos SN (2016). An integrated approach to correction for off-resonance effects and subject movement in diffusion MR imaging. Neuroimage.

[34] Gorgolewski KJ (2016). The brain imaging data structure, a format for organizing and describing outputs of neuroimaging experiments. Sci. Data.

[35] Tong Q (2020). figshare.

[36] National Electrical Manufacturers Association. *Determination of signal-to-noise ratio (SNR) in diagnostic magnetic resonance imaging*. Report No. MS 1-2008 (R2014) (National Electrical Manufacturers Association, 2008).

[37] Henkelman RM (1985). Measurement of signal intensities in the presence of noise in MR images. Medical physics.

[38] Smith RE, Tournier JD, Calamante F, Connelly A (2013). SIFT: spherical-deconvolution informed filtering of tractograms. Neuroimage.

[39] Tong Q, He H (2020). figshare.

